# The impact of industrial robot adoption on corporate green innovation in China

**DOI:** 10.1038/s41598-023-46037-8

**Published:** 2023-10-31

**Authors:** Lin Liang, Liujie Lu, Ling Su

**Affiliations:** 1 College of Business, Xinjiang University, No. 499 Northwest Road, Urumqi, Xinjiang China; 2 Business School, University of International Business and Economics, No. 10 Huixin East Street, Beijing, China; 3 College of Accounting, Xinjiang University of Finance and Economics, No. 320 Beijing South Road, Urumqi, Xinjiang China

**Keywords:** Environmental economics, Sustainability

## Abstract

Green innovation plays a crucial role in transforming economic models and achieving sustainable development in enterprises. As an important embodiment of artificial intelligence technology, how industrial robots can effectively promote the green transformation of enterprises has become an important issue. This paper examines the impact and mechanisms of industrial robot adoption on corporate green innovation, as well as its heterogeneous effects. Using data from Chinese listed companies from 2007 to 2019, we find that industrial robot adoption has a significant positive impact on corporate green innovation, enhancing both its quantity and quality. Furthermore, our mechanism study reveals that industrial robot adoption can promote corporate green innovation by improving productivity and environmental management capabilities. Additionally, we investigate the moderating effects of various factors and conclude that the positive impact of industrial robot adoption on green innovation is more pronounced among the state-owned enterprises, enterprises with the intense market competition, as well as enterprises located in regions with higher carbon emissions intensity. This paper contributes to enrich the research on industrial robots and corporate green innovation, and provides a reference to improve environmental management and achieve a low-carbon economy in emerging markets.

## Introduction

In recent years, the growing recognition of the severe environmental problems has underscored the need for green innovation as a solution to environmental pollution and a pathway to sustainable economic growth^1^. As the micro subjects of economic activities, enterprises play a significant role in independent innovation and green technological development, and are critical to ecological and environmental management^2^. The integration of green innovation into enterprise operations not only facilitates energy conservation and pollution reduction, which in turn reduces environmental costs and improves their environmental protection capabilities^3,4^; but also enables enterprises to create new market demands by producing environmentally products and obtaining sustainable production models. However, enterprises are currently faced with both financial and technological issues when it comes to green innovation: the higher risk and larger investment of green innovation cause serious financing constraints^5^. Therefore, it is critical to investigate how to promote environmental management and green innovation in enterprises. Artificial intelligence technology, as an essential driving force for green innovation, can effectively overcome the challenges encountered by enterprises during their pursuit of green innovation. Given the pivotal role of artificial intelligence and intelligent manufacturing, there is an urgent need to investigate whether industrial robots can enhance green innovation and facilitate the environmentally friendly transformation of manufacturing enterprises.

Enterprises wield considerable influence in terms of their contribution to environmental pollution, and green innovation is a critical step towards resolving the challenge of synergistic economic and environmental development. Factors that impact corporate green innovation have been classified into two principal groups, namely the external environment and corporate governance, according to prior research. Regarding external environmental factors, environmental regulatory policies^6–9^, financial development^10,11^, and stakeholder behavior^12,13^ play a crucial role in fostering positive outcomes for corporate green innovation. For environmental regulation, most of the studies have been based on neoclassical economic theory and Porter's hypothesis, but there remains a lack of consensus on whether environmental regulation can contribute positively to the advancement of green innovation. With regards to internal corporate governance, ownership structure^5^, executive characteristics^14,15^ and board characteristics^16,17^ have been identified as potential drivers of green innovation. Nevertheless, the existing literature has relatively little consideration of the potential impacts of artificial intelligence on corporate green innovation, particularly regarding industrial robot adoption. As industrial robots become more intelligent and automated, their specific role in advancing green technology in enterprises and facilitating the transformation of green production needs further exploration. Therefore, this paper contributes to deepening research on industrial robots and corporate green innovation while unveiling various factors impacting it from the perspective of artificial intelligence. Additionally, it facilitates the corporate green innovation capabilities and global energy and environmental governance.

From Smith and Ricardo's "Theory of Value" to Marx's notion of the "Industrial Reserve Army" to Schumpeter's theory of technological innovation and economic cycles, there has been no consensus on whether new technologies lead to unemployment. Currently, research on the economic consequences of industrial robot adoption has primarily focused on the labor market. Some scholars argue that industrial robots, as a technological advancement with a bias towards skills, may result in the displacement of low-skilled labor while generating a greater demand for high-skilled labor^18,19^. However, others suggest that industrial robots can create new employment, attract high-skilled employees, and improve the labor structure^20–23^. Overall, the focus of the existing literature primarily revolves around the analysis of how industrial robots affect the macro labor market, with little attention given to the impact of micro-firm innovation decisions. In particular, the mechanisms of green innovation in enterprises directly associated with environmental protection have received little attention, and relevant empirical studies are scarce. Furthermore, the limited availability of micro-level data on robot adoption has led existing research to primarily concentrate on the national or industry level, resulting in insufficient micro-level research^24,25^. Additionally, the impact of industrial robot adoption in China is expected to be more extensive and representative than in other nations and industries due to the scale, population, resource consumption, and carbon emissions of the Chinese manufacturing industry^26^. Consequently, by delving into the correlation between industrial robots and green innovation, this study enhances the current research on the economic consequences of industrial robot adoption and offers valuable insights for facilitating corporate technology upgrading and green industry transformation in emerging market countries.

This paper has the potential to contribute to research in the following areas: Firstly, this study examines how industrial robot adoption affects green innovation, taking into account the viewpoint of artificial intelligence. It broadens the scope of research on the factors that influence green innovation from the perspective of artificial intelligence and provides valuable insights on how to facilitate corporate green innovation and global green environmental performance. Secondly, this paper further clarifies the impact of artificial intelligence in promoting corporate green transformation through industrial robot adoption and expands the relevant research on the role of industrial robots in driving green innovation. As an important subject of industrial robot adoption, enterprises occupy a pivotal position in the advancement of green innovation. It provides valuable insights for large-scale industrial robot adoption and accelerates the green transformation through technology. Finally, this study analyzes how industrial robots enhance corporate green innovation and clarifies the pathways through the "productivity effect" and "environmental management effect". By employing a theoretical model and investigating mediating effects, it establishes the complex relationship between industrial robots and green innovation, thereby shedding light on the previously elusive "black box" and providing a conceptual framework for exploring robot adoption.

The remaining sections of the current work are structured as follows. Section "Literature review" shows the literature review, Section "Hypothesis development" demonstrates the hypotheses. Section "Methodology and data" focuses on the methods and data. Section "Empirical analysis and results" shows the empirical analysis and results. Section "Mechanism tests" shows the mechanism tests. Section "Heterogeneity analysis" analyzes heterogeneity. Section "Discussion" dicusses the results. Finally, section “Conclusion and policy implications” concludes the research and provides policy implications.

## Literature review

This study explores the impact of industrial robot adoption on corporate green innovation by reviewing two different streams of literature: the economic consequences of industrial robot adoption and the determinants of green innovation.

### Economic consequences of industrial robot adoption

With the rapid progress of artificial intelligence and automation technology, industrial robots have become a significant technological advancement, enhancing the market competitiveness of manufacturing enterprises. They have the potential to replace traditional labor engaged in simple, repetitive, and low-skilled work, gaining a comparative advantage and supporting the intelligent upgrading of the manufacturing industry. There is no consensus on how industrial robot adoption will affect the labor structure, but there are two basic perspectives as follows: on the one hand, industrial robot adoption can result in less demand for labor, playing a "substitution effect." On the other hand, industrial robot adoption can create new employment, attract high-skilled employees, and improve the labor structure, playing a "creation effect."

Firstly, concerning the "substitution effect" of industrial robots, Acemoglu and Restrepo^27^ prove that industrial robot adoption has the potential to negatively impact employment and wages. Their findings indicate that for every additional industrial robot per thousand employees, there is a corresponding decrease of 0.2% in the employment rate and a reduction of 0.42% in wages. Brambilla et al.^28^ show that industrial robot adoption has engendered a consequential deterioration in key metrics of the labor market, notably evidenced by amplified unemployment rates and the accentuation of the informalization of the workforce. Jung and Lim^19^ suggest that industrial robot adoption has led to a decline in the utilization of low-skilled labor, thereby curbing employment growth. Acemoglu and Restrepo^18^ state that automation will consistently decrease the proportion of labor and may even decrease the need for labor.

Secondly, in relation to the "creation effect" of industrial robots, Koch et al.^23^ discover that industrial robot adoption leads to a significant increase in output by approximately 20–25% within a span of four years. Additionally, their findings indicate a reduction in labor costs by 5–7% and an increase in employment by 10%. Graetz and Michaels^20^ also emphasize the positive influence of industrial robot adoption on labor productivity and income levels. Specifically, for every 1% increase in industrial robot adoption, the labor productivity of enterprises increases by 0.36%. Bonfiglioli et al.^22^ show that industrial robot adoption improves the demand for high-skilled employees while having little impact on total sales. Balsmeier and Woerter^21^ further discover that the majority of the new jobs created by industrial robots are meant to supplement high-skilled workers.

Besides the effect on labor, some studies provide evidence of its impact on energy efficiency and carbon reduction. Huang et al.^29^ discover that industrial robot adoption can significantly enhance corporate energy efficiency. Additionally, industrial robot adoption can increase corporate productivity and thus improve energy efficiency. Liu and Li^30^ find that industrial robot adoption increases productivity, optimizes factor structures, and introduces technical innovation into production. As a result, energy efficiency is improved while carbon intensity is reduced^25^.

### Influencing factors of green innovation

The current research focuses on the influencing factors of green innovation based on macro factors, stakeholder characteristics, and corporate governance.

From a macro perspective, existing research has predominantly concentrated on environmental regulation. However, there is little consensus regarding how it affects corporate green innovation. One perspective is based on classical economic theory^6^, suggesting that environmental regulation can escalate the expense of pollution, create a "crowding effect" on funding, and pose obstacles to the progress of green innovation. Zhang^31^ emphasizes that environmental regulation can exert a detrimental influence upon the green innovation endeavors of heavily polluting corporations. Greenstone et al.^8^ find that air quality regulations significantly reduce productivity, thereby restraining corporate green innovation. The other perspective is based on the Porter hypothesis^7^, suggesting that effective environmental regulation can incentivize enterprises to adopt green innovation practices and enhance their competitive edge, resulting in what is referred to as the "innovation compensation" effect. Guo et al.^32^ indicate a substantial enhancement of corporate green innovation due to environmental regulation. Peng et al.^33^ further discover that environmental regulation can contribute to the overall encouragement and advancement of green innovation activities.

At the stakeholder characteristics level, existing studies have primarily concentrated on consumers and suppliers. Zhang and Zhu^12^ reveal that consumer pressure exerts greater benefits for the innovation of green products compared to regulatory pressure. Conversely, regulatory pressure demonstrates a more pronounced impact on the innovation of green processes in contrast to consumer pressure. Yang and Lin^13^ provide evidence that the relationships between partners within the supply chain significantly affect green innovation performance. Consequently, it is crucial to consider supply chain partners as an integral part of green innovation strategies.

At the corporate governance level, existing studies have primarily concentrated on equity structure, executive characteristics, and board characteristics. Amore and Bennedsen^5^ indicate that ineffective corporate governance leads to fewer green patents and a decrease in green innovation. He and Jiang^16^ suggest that female representatives on the board are systematically connected to green innovation. The more women on the board, the more likely green product innovation. Quan et al.^14^ further find that the foreign experience of CEOs enhances green innovation by improving environmental ethics and general competence. Bin Yousaf et al.^17^ discover that the enhancement of corporate green innovation is facilitated by board capital. This role is dominated by absorptive capacity.

### Literature summary

First, existing studies have predominantly concentrated on exploring the influence of industrial robot adoption on the macro labor market and employment. However, few studies have analyzed how industrial robot adoption can affect the green development of micro-enterprises, especially regarding green innovation. Enterprises, as the carriers of industrial robot adoption, should be guided by the principles of green innovation, integrating the concepts of sustainable development into their research and development, manufacturing, and sales processes. Therefore, this research analyzes whether industrial robot adoption can enhance green innovation, offering valuable theoretical insights into the environmental management implications of adopting industrial robots in terms of corporate green innovation.

Second, the current literature investigates the factors that influence green innovation, including macro factors, stakeholder characteristics, and corporate governance. However, previous studies rarely analyze the role of industrial robot adoption in green innovation from the standpoint of artificial intelligence and automation technologies. And it has largely ignored the dynamic adjustment process of artificial intelligence driving green innovation. Artificial intelligence serves as a significant catalyst for promoting sustainable corporate growth and driving the transformation of manufacturing industries. In light of this gap in existing studies, our research analyzes how industrial robot adoption facilitates green innovation.

## Hypothesis development

Industrial robot adoption has generated significant transformations in the economic and social development of China, fostering green innovation in enterprises. Technological progress has yielded positive externalities, leading to improved energy efficiency, reduced energy consumption, and fostering green innovation in enterprises^34,35^. Industrial robots, as a prominent example of artificial intelligence technology, directly impact corporate green innovation by improving production efficiency and environmental management capabilities. Firstly, with the continuous development of digital technology, industrial robots will gradually replace repetitive and traditional labor, improving the production efficiency of enterprises. Based on this, they can promote the optimization and recombination of production factors^36,37^, resulting in enhanced energy efficiency and a decrease in carbon intensity, thereby promoting green innovation. Secondly, industrial robot adoption will shift the labor structure from skill-based to knowledge-based, resulting in higher demands for labor skills^21,23^. High-skilled employees have the capacity to facilitate the application of green technology, generate spillover effects of green technology, build green innovation networks within the enterprise, and improve green technology innovation. Finally, industrial robot adoption can quickly identify pollution sources, realize intelligent environmental monitoring, and enable enterprises to master advanced clean technologies^38,39^. Saldanha et al.^40^ indicate that clean technology can enhance the operational efficiency of enterprises in utilizing scarce resources, reduce their dependence on traditional energy, and promote environmental management. Therefore, industrial robot adoption can promote green innovation by achieving clean production and improving their environmental management. Therefore, we put forth the following research hypotheses.

### Hypothesis 1

Industrial robot adoption promotes corporate green innovation.

Industrial robots are an important application of artificial intelligence that can expand the range of products and services in company operations^41^. They can optimize production processes, improve efficiency, and exert a favorable influence on green innovation. Here are the specific factors contributing to this phenomenon: firstly, enterprises using industrial robots can acquire and absorb green technologies and knowledge, enable them to integrate internal and external knowledge, enhance the spillover effect of green technology, and promote productivity^42^. In this way, enterprises can successfully develop green products, resulting in reduced energy consumption and decreased dependence on traditional energy sources^43,44^. In turn, this promotes the transition to green manufacturing and enhances corporate green innovation. Secondly, the adoption of industrial robots has partially automated and mechanized the manufacturing process, improving production processes and productivity. Furthermore, innovation in production processes promotes green technology innovation by lowering energy consumption, cutting manufacturing costs^45^, and increasing energy efficiency. Finally, industrial robot adoption can improve productivity, reduce production and operation costs, and promote the reduction of product prices, leading to an increase in product demand and the expansion of output scale^46,47^. Moreover, this effect will generate enough cash flow to improve corporate profitability and core competitiveness, which can serve as crucial financial support for promoting green innovation. Therefore, we put forward the subsequent research hypotheses.

### Hypothesis 2

Industrial robot adoption promotes corporate green innovation by improving productivity.

Technological advances can improve energy efficiency, conserve energy consumption, and reduce carbon dioxide emissions. Industrial robots are the latest achievement in information technology and have advantages such as high efficiency, high precision, and sustainability. They have the potential to positively impact the environmental management capacities of enterprises by enabling cleaner production, increasing energy efficiency, and reducing waste^48,49^. Firstly, robots enable intelligent manufacturing and real-time monitoring of energy consumption and pollution during product production. With standardized management, they can reduce excessive emissions caused by inefficient energy consumption^50^, thus realizing clean production, and enhancing corporate environmental management capacities. Enterprises can now leverage novel technology to undertake and foster green innovation activities^47^. Secondly, industrial robot adoption makes production processes more data-driven, transparent, and intelligent^38^. This results in the optimization of production factors, improving production processes, and effectively identifying and solving standardization problems during production^51,52^. This can improve energy efficiency, reduce carbon intensity, enhance environmental management capacities, and promote corporate green innovation. Finally, industrial robot adoption can help enterprises integrate internal and external resources in a broader time and space range, providing a resource basis for green innovation^53^. Specifically, through information monitoring and exchange, industrial robots can reorganize and allocate resources^54^, generate high-value composite resources, and increase the effectiveness of allocating green resources. This reduces waste and repeated consumption of resources^48^ and enhances environmental management capacities, promoting corporate green innovation. Therefore, we put forward the subsequent research hypotheses.

### Hypothesis 3

Industrial robot adoption promotes corporate green innovation by improving environmental management capacities.

## Methodology and data

### Sample and data sources

We choose A-share listed manufacturing companies as our research subjects for the period from 2007 to 2019. This selection is based on the widespread adoption of industrial robots in China since 2007, and the latest available industrial robot data is only updated through 2019. Our source of industrial robot data is the International Federation of Robotics (IFR), which surveys global robot manufacturers. This database provides authoritative data on the applications, industries, and types of robots used worldwide. To ensure that the listed company data matches the industrial robot data, we manually match a two-digit code in Chinese manufacturing industry with the industry codes provided by IFR.

We obtain green innovation data from Chinese Research Data Services (CNRDS) and financial data from the China Stock Market & Accounting Research Database (CSMAR). The process of data selection is as follows: (1) exclusion of the non-manufacturing listed samples; (2) exclusion of samples labeled as Special Treatment (ST) and Particular Transfer (PT); (3) exclusion of samples with owner's equity below 0; (4) exclusion of samples with incomplete or missing data; and (5) application of winsorization to all continuous variables at the 1% and 99% quantile levels. After this selection process, our final sample comprises 14,831 observations.

### Variable constructions

#### Dependent variable

Most current studies categorize green patents into two groups: green invention patents and green utility patents^55^. Green invention patents are characterized by a higher level of inventiveness and technicality, whereas green utility patents primarily focus on protecting the shape and structure of the product. Drawing upon Bai et al.^56^, we define the quantity of green innovation (GIS) as the natural logarithm of the number of green patent applications plus one for listed companies. The quality of green innovation (GIZ) is measured by the natural logarithm of the number of green invention patent applications plus one for listed companies.

#### Independent variable

Drawing upon Acemoglu and Restrepo^27^, we construct Chinese industrial robot penetration. To deconstruct the impacts of exogenous technology (robots) from a macroscopic to a firm-level perspective, we employ the "Bartik variable"^57^. Since industrial robots have both technological and capital characteristics, manufacturing enterprises with a higher percentage of fixed assets and intangible assets have a higher penetration of industrial robots. We calculate the penetration of industrial robots and add weights to deconstruct it from the industry level to the enterprise level, which is a more accurate and reliable measure.

#### Control variables

Drawing upon Bin Yousaf et al.^17^, we comprise the following control variables to mitigate the influence of various factors other than industrial robot adoption on green innovation as much as possible. First, the scale of an enterprise stands as a pivotal determinant influencing green innovation. The expansion of scale stimulates enterprises to engage in research and development initiatives more proactively, thereby fostering the sharing of green innovation resources among diverse enterprises and the elevation of green innovation^58^. As such, we primarily measure a company's scale through its total market value and the number of employees. Second, the financial condition of an enterprise is a significant influencing factor in corporate investment decisions, reflecting the company's profitability and efficiency in capital utilization. Profitability not only contributes to stimulating a company's research and development investments but also to the cultivation of green innovation activities. Therefore, we select indicators such as LEV, GROW, OCF, LOSS, and OTHREC as control variables related to green innovation^59–61^. Third, the age of an enterprise exerts a discernible influence on new product development, research and development investments, as well as outcomes in the realm of green innovation^62^. Fourth, green technologies and practices might necessitate greater investments and time. Enterprises with highly concentrated equity ownership may be more inclined to engage in long-term investments in green innovation, thereby translating research and development efforts into product innovation^63^. Consequently, this study employs the proportion of shares held by the largest shareholder as a metric to gauge the degree of equity concentration. Moreover, both the year and industry fixed effects are also controlled. Table 1 displays the variable definitions.

**Table 1:** Variable definitions.

Variables	Definition
ROBOT	The penetration of firms’ industrial robots
GIS	The natural logarithm of the number of green patent applications plus one
GIZ	The natural logarithm of the number of green invention patents applications plus one
SIZE	The natural logarithm of total market value
LEV	Total liabilities/total assets
GROW	Income growth rate
OCF	Operating cash flow/total assets
AGE	The logarithm of the company's listing age plus one
LOSS	If the net profit is less than 0, the value is 1, and otherwise, it is 0
SHRCR	The shares held by the largest shareholder/Total shares
OTHREC	Other receivables/total assets
STAFF	The logarithm of the number of employees plus one
INDUSTRY	Industry dummy variable
YEAR	Year dummy variable

### Model specification

To examine the effect of industrial robot adoption on green innovation, we employ the following regression models:

1 $${GIS/GIZ}_{i,t+1}={\beta }_{0}+{\beta }_{1}{ROBOT}_{i,t}+\sum {CONTROLS}_{i,t}+{\varepsilon }_{i,t}$$

where GIS/GIZ represents corporate green innovation, ROBOT shows the penetration of industrial robots in enterprises, CONTROLS encompasses the control variables, and ε_i,t_ denotes the random disturbance. Additionally, to address the issue of delayed green innovation and mitigate the potential endogeneity concern, we introduce a one-year lag for all independent variables. In accordance with the model, this study places emphasis on β1. The significance of a positive value for β1 would imply that industrial robot adoption enhances green innovation, thereby confirming hypothesis 1.

## Empirical analysis and results

### Descriptive statistics and correlation analysis

Descriptive statistics are presented in Table 2. The mean value for ROBOT is 1.9059, ranging from 0 to 6.5981, with a standard deviation of 1.5338. These results indicate that industrial robots are already widely used in enterprises, but a considerable number of enterprises have low robot adoption. Regarding green innovation capabilities, GIS has an average value of 0.9552, while GIZ has a mean of 0.6316, indicating variations in green innovation capabilities across different enterprises. Control variables show a mean value of 22.6333 for SIZE and 0.5828 for LEV. The average values of GROW, OCF, and AGE are 0.1575, 0.0485, and 2.8629, respectively. LOSS has a mean value of 0.1051, demonstrating that 10.51% of enterprises experience negative net profit. SHRCR, OTHREC, and STAFF have average values of 0.3392, 0.0130, and 7.8044, respectively.

**Table 2:** Descriptive statistics.

Variables	N	Mean	SD	Min	P25	P50	P75	Max
ROBOT	14,831	1.9059	1.5338	0.0000	0.6665	1.6052	2.7797	6.5981
GIS	14,831	0.9552	1.1915	0.0000	0.0000	0.6931	1.6094	4.6634
GIZ	14,831	0.6316	0.9806	0.0000	0.0000	0.0000	1.0986	4.1897
SIZE	14,831	22.6333	1.0270	20.5762	21.9096	22.5187	23.2169	25.6261
LEV	14,831	0.5828	0.1927	0.1341	0.4356	0.5874	0.7347	0.9465
GROW	14,831	0.1575	0.3384	-0.4896	-0.0193	0.1082	0.2593	2.1078
OCF	14,831	0.0485	0.0668	-0.1498	0.0092	0.0459	0.0881	0.2392
AGE	14,831	2.8629	0.3080	1.7918	2.6391	2.8904	3.0910	3.4657
LOSS	14,831	0.1051	0.3067	0.0000	0.0000	0.0000	0.0000	1.0000
SHRCR	14,831	0.3392	0.1400	0.0905	0.2306	0.3208	0.4286	0.7187
OTHREC	14,831	0.0130	0.0190	0.0002	0.0029	0.0068	0.0146	0.1243
STAFF	14,831	7.8044	1.1206	5.1180	7.0326	7.7324	8.5184	10.8176

Table 3 illustrates the correlation coefficients between the variables. The top right section displays the Spearman correlation coefficients, while the bottom left section displays the Pearson correlation coefficients. The Spearman correlation coefficients indicate a significant correlation between the coefficients of ROBOT and GIS/GIZ, supporting hypothesis 1. Additionally, the coefficients of GIS/GIZ show significantly positive correlations with SIZE, GROW, AGE, OTHREC, STAFF, while they have significantly negative correlations with LEV, OCF, LOSS, SHRCR. The Pearson correlation coefficients further demonstrate a noteworthy association between the ROBOT coefficients and the coefficients of GIS/GIZ, providing support for hypothesis 1. Moreover, the coefficients of GIS/GIZ exhibit substantial positive correlations with SIZE, GROW, AGE, and STAFF, whereas they display notable negative correlations with LEV, OCF, and LOSS.

**Table 3:** Correlation coefficients.

	ROBOT	GIS	GIZ	SIZE	LEV	GROW	OCF	AGE	LOSS	SHRCR	OTHREC	STAFF
ROBOT	1	0.4166***	0.3867***	0.5419***	− 0.2166***	0.0483***	0.0607***	0.2791***	0.0054	0.0196**	− 0.0706***	0.4141***
GIS	0.4637***	1	0.8831***	0.3870***	− 0.1910***	0.0693***	− 0.0218***	0.0875***	− 0.0298***	− 0.0187**	0.0911***	0.3174***
GIZ	0.4396***	0.9242***	1	0.3771***	− 0.1730***	0.0637***	− 0.0142*	0.0819***	− 0.0325***	− 0.0165**	0.0910***	0.2941***
SIZE	0.5615***	0.4487***	0.4445***	1	− 0.3565***	0.0759***	0.1029***	0.2011***	− 0.0623***	0.0935***	− 0.0052	0.6773***
LEV	− 0.2420***	− 0.2102***	− 0.1911***	− 0.3591***	1	0.0074	0.1554***	− 0.0560***	− 0.1931***	− 0.0374***	− 0.1947***	− 0.4286***
GROW	0.0340***	0.0576***	0.0522***	0.0773***	− 0.0135	1	0.0536***	− 0.0950***	− 0.2711***	0.0093	− 0.0059	0.0237***
OCF	0.0493***	− 0.0202**	− 0.0128	0.1208***	0.1652***	0.0171**	1	0.0223***	− 0.1948***	0.0765***	− 0.1786***	0.1367***
AGE	0.2642***	0.0838***	0.0843***	0.2019***	− 0.0770***	− 0.0704***	0.0217***	1	0.0336***	− 0.1221***	− 0.0103	0.1059***
LOSS	0.0028	− 0.0318***	− 0.0337***	− 0.0609***	− 0.2086***	− 0.2133***	− 0.1956***	0.0378***	1	− 0.0696***	0.0833***	− 0.0467***
SHRCR	0.0356***	− 0.0058	− 0.0058	0.1302***	− 0.0431***	0.0153*	0.0814***	− 0.1228***	− 0.0641***	1	− 0.1278***	0.1526***
OTHREC	− 0.0531***	0.0078	0.0121	− 0.0259***	− 0.1499***	− 0.0398***	− 0.1496***	0.0288***	0.1285***	− 0.1210***	1	0.0264***
STAFF	0.4643***	0.3759***	0.3576***	0.7206***	− 0.4204***	0.0176**	0.1417***	0.0955***	− 0.0522***	0.1717***	− 0.0487***	1

### Baseline regressions

The findings for the quantity of green innovation are exhibited in columns (1)–(3) of Table 4, while the outcomes for its quality are displayed in columns (4)–(6). In columns (1) and (4), the regression results control only for the industry and year fixed effects without including any control variables. Columns (2) and (5) incorporate additional control variables but do not include the year and industry fixed effects. Columns (3) and (6) display results that consider both control variables and control for the industry and year fixed effects. The estimated outcomes in columns (1)–(3) consistently exhibit positive and significant coefficients for ROBOT at the 1%. This suggests that industrial robot adoption substantially boosts the quantity of green innovation. Examining columns (4)–(6) reveals significantly positive coefficients for ROBOT at the 1%, indicating a remarkable improvement in the quality of green innovation resulting from industrial robot adoption. Therefore, industrial robot adoption can enhance both the quantity and quality of green innovation, supporting hypothesis 1.

**Table 4:** Baseline regressions.

Variables	(1)	(2)	(3)	(4)	(5)	(6)
	GIS_t+1_	GIS_t+1_	GIS_t+1_	GIZ_t+1_	GIZ_t+1_	GIZ_t+1_
ROBOT	0.2299*** (14.3296)	0.1654*** (11.3355)	0.1713*** (10.4318)	0.2175*** (14.7819)	0.1515*** (11.0972)	0.1743*** (11.6413)
SIZE		0.2091*** (9.9814)	0.1964*** (7.4878)		0.1639*** (8.8609)	0.1567*** (6.6288)
LEV		0.2198** (2.2851)	0.1664* (1.7422)		0.2018** (2.3955)	0.1682** (2.0056)
GROW		0.0067 (0.3278)	0.0041 (0.1992)		− 0.0210 (− 1.1763)	− 0.0165 (− 0.9167)
OCF		− 0.1141 (− 1.0170)	− 0.0672 (− 0.6028)		− 0.0632 (− 0.6657)	− 0.0315 (− 0.3324)
AGE		0.3796*** (5.5476)	− 0.2198 (− 1.3436)		0.1679*** (2.7510)	− 0.2621* (− 1.7893)
LOSS		− 0.0899*** (− 3.8236)	− 0.0892*** (− 3.7445)		− 0.0718*** (− 3.5987)	− 0.0719*** (− 3.6066)
SHRCR		0.2189 (1.3094)	0.1860 (1.1774)		0.2301 (1.5647)	0.1751 (1.2266)
OTHREC		− 0.0239 (− 0.0528)	0.2820 (0.6291)		0.1477 (0.3774)	0.4247 (1.0888)
STAFF		0.1178*** (4.9232)	0.1182*** (4.9969)		0.0998*** (4.8763)	0.0885*** (4.3084)
Constant	0.1017 (0.5720)	− 6.2033*** (− 13.1180)	− 4.8073*** (− 7.2438)	− 0.0342 (− 0.2243)	− 4.7470*** (− 11.4356)	− 3.7380*** (− 6.2889)
Observations	14,831	14,831	14,831	14,831	14,831	14,831
R-squared	0.2218	0.2253	0.2381	0.1968	0.1962	0.2107
Industry FE	Yes	No	Yes	Yes	No	Yes
Year FE	Yes	No	Yes	Yes	No	Yes

### Robustness test

#### IV estimation

To address endogeneity concerns, this study employs instrumental variable (IV) estimation as a robustness test. Following Acemoglu and Restrepo^27^, the penetration of industrial robots in the US is used as an instrumental variable to test the robustness using the two-stage least squares method (2SLS). The choice is based on two primary considerations. First of all, the development degree and application level of industrial robots in the United States are similar to those of the same period in China, and the development trend is relatively close; Secondly, the United States holds a leading position in terms of robot development globally, which can represent the technological development trend of the industry. Moreover, the effects of industrial robots on the advancement of corporate green innovation in the United States should only reflect exogenous technological progress and spillover. Thus, industrial robot penetration in the US aligns with the correlation and exogeneity requirements of the IV estimation.

The findings of the IV regression are displayed in Table 5. In column (1), the outcomes of the first-stage regression are displayed, indicating a statistically significant positive coefficient for USROBOT_IV at the 5% level. This suggests a strong correlation between U.S. robots and Chinese industrial robots. Columns (2)–(3) exhibit the findings of the second-stage regression. The outcomes reveal statistically significant coefficients for ROBOT, reaching a significance level of 5%. They indicate that green innovation is still significantly impacted by industrial robot adoption, further mitigating potential endogenous problems.

**Table 5:** IV estimation.

Variables	(1)	(2)	(3)
	First stage	Second stage	Second stage
	ROBOT	GIS_t+1_	GIZ_t+1_
USROBOT_IV	0.0001** (2.4243)		
ROBOT		1.4760** (1.9656)	2.5493** (2.3687)
Controls	Yes	Yes	Yes
Industry FE	Yes	Yes	Yes
Year FE	Yes	Yes	Yes
Observations	14,831	14,831	14,831

#### Change variables and models

To ensure the credibility of the findings, we also make adjustments to the calculation method for dependent and independent variables. Firstly, we modify the metric for the independent variable to measure industrial robot penetration by the number of operating robots (ROBOT2). The estimation outcomes, presented in Columns (1) and (2) of Table 6, still demonstrate significantly positive coefficients for ROBOT2 at the 1% significance level. Secondly, we modify the measurement of the dependent variable. The quantity of green innovation (GIGS) is the natural logarithm of the number of green patent authorizations plus one for listed companies. The quality of green innovation (GIGZ) is the natural logarithm of the number of green invention patent authorizations plus one for listed companies. The outcomes are exhibited in Columns (3) and (4) of Table 6. The coefficients associated with ROBOT remain positively significant at the 1% level. Thirdly, the regression model is further replaced with the Tobit model. The estimation results, depicted in Columns (5) and (6) of Table 6, reveal that the coefficients of ROBOT are statistically significant at the 1% level. Therefore, the outcomes show that the baseline regression findings are robust after modifying variables and models.

**Table 6:** Change variables and models.

Variables	(1)	(2)	(3)	(4)	(5)	(6)
	GIS_t+1_	GIZ_t+1_	GIGS_t+1_	GIGZ_t+1_	GIS_t+1_	GIZ_t+1_
ROBOT2	0.1571*** (9.5457)	0.1621*** (10.8309)				
ROBOT			0.1869*** (12.3834)	0.1404*** (11.3934)	0.0830*** (4.2462)	0.1121*** (5.3199)
Constant	− 4.6607*** (− 6.9162)	− 3.5682*** (− 5.9163)	− 3.2925*** (− 5.6170)	− 1.1657*** (− 2.6433)	− 12.6670*** (− 20.7708)	− 13.5023*** (− 20.6680)
Controls	Yes	Yes	Yes	Yes	Yes	Yes
Industry FE	Yes	Yes	Yes	Yes	Yes	Yes
Year FE	Yes	Yes	Yes	Yes	Yes	Yes
Observations	14,831	14,831	14,831	14,831	14,831	14,831
R-squared	0.2367	0.2091	0.2051	0.1328	0.1249	0.1837

#### Persistent test

To further explore the persistence of industrial robot adoption, the independent variable is delayed by two periods and three periods. The outcomes presented in Table 7 demonstrate that the coefficients associated with ROBOT remain positively significant at the 1% level, illustrating the reliability of the research findings.

**Table 7:** Persistent test.

Variables	(1)	(2)	(3)	(4)
	GIS_t+2_	GIZ_t+2_	GIS_t+3_	GIZ_t+3_
ROBOT	0.1302*** (7.2907)	0.1455*** (8.8569)	0.1071*** (5.3268)	0.1260*** (7.0163)
Constant	− 2.7602*** (− 3.8550)	− 2.0654*** (− 3.2225)	− 1.3978* (− 1.7883)	− 1.0037 (− 1.4742)
Controls	Yes	Yes	Yes	Yes
Industry FE	Yes	Yes	Yes	Yes
Year FE	Yes	Yes	Yes	Yes
Observations	12,437	12,437	10,522	10,522
R-squared	0.1914	0.1692	0.1627	0.1415

## Mechanism tests

In the above analysis, we have shown that industrial robot adoption can enhance corporate green innovation. This subsection delves deeper into exploring the underlying factors that contribute to this positive relationship. Building upon the hypothesis development analysis, we put forth three potential mechanisms through which industrial robot adoption fosters green innovation.

### Mediating effect of productivity

We use capital productivity and labor productivity to measure productivity. Based on the relevant literature^64,65^, we employ the ratio of net value of fixed assets to the number of employees to evaluate capital productivity (CPE). According to Spaliara^66^, the ratio of operating income to the number of employees is employed to count labor productivity (SPE). The mediating effect of productivity is depicted in Table 8, showcasing the coefficient of ROBOT on CPE at a significant level of 1%, measuring 0.2046. Additionally, the coefficient of ROBOT on SPE stands at 0.0266 and exhibits significance, being positive at the 5% level. Overall, industrial robot adoption can promote green innovation through improving productivity.

**Table 8:** The mediating effect of productivity and environmental management capability.

Variables	(1)	(2)	(3)
	CPE_t+1_	SPE_t+1_	EPI_t+1_
ROBOT	0.2046*** (15.2128)	0.0266** (2.5654)	0.3857*** (2.6129)
Constant	11.3382*** (23.3958)	10.5614*** (24.6191)	1.8046 (0.3070)
Controls	Yes	Yes	Yes
Industry FE	Yes	Yes	Yes
Year FE	Yes	Yes	Yes
Observations	14,831	14,831	14,831
R-squared	0.2372	0.3394	0.0379

### Mediating effect of environmental management capability

Referring to Zhang et al.^67^, we use environmental investments to measure environmental management capability. We manually collect the expenditure in the corporate annual reports related to environmental investments, which are in the detailed items “construction in progress” (including waste gas treatment; energy-saving, water-saving and electricity-saving; desulfurization projects, denitrification projects, and nitrogen projects, etc.), and “administrative expense” (including sewerage fees and greening fees, etc.). Then, environmental management capability (EPI) is represented by the natural logarithm of environmental investments. Column (3) in Table 8 depicts the mediating effect of environmental management capability. The coefficient of ROBOT on EPI is significant at the 1% level. Overall, industrial robot adoption can enhance green innovation by promoting environmental management capability.

## Heterogeneity analysis

We have previously proven the positive association between industrial robot adoption and the advancement of corporate green innovation. Nevertheless, a subsequent question emerges: do distinctions exist in how industrial robot adoption affects green innovation across enterprises with diverse characteristics and geographical regions? To comprehensively address this question, it becomes imperative to conduct in-depth analysis and engage in detailed discussions.

### The heterogeneity of corporate ownership

Distinct forms of corporate ownership exhibit variations in corporate goals and strategic directions. Firstly, in addition to financial benefits, Chinese state-owned enterprises (SOEs) frequently take on social responsibilities, including maintaining social stability and protecting the environment, which further motivates their proactive engagement in green innovation activities^68^. Secondly, when governments formulate policies to support green growth, SOEs are more likely to benefit from them and promote green development^69^. Therefore, we expect that in SOEs, industrial robot adoption can exert a more pronounced influence on green innovation.

We examine the heterogeneity of corporate ownership. If the enterprise is a SOE, the value is 1, otherwise it is 0. The interaction term between SOE and industrial robot adoption is constructed. The outcomes in Columns (1) and (2) of Table 9 illustrate the heterogeneity of corporate ownership. Notably, the interaction term (ROBOT*SOE) exhibits statistically significant coefficients at a significance level of 5%. The outcomes demonstrate that industrial robot adoption causes a pronounced effect on improving green innovation in SOEs.

**Table 9:** Heterogeneity analysis results of corporate ownership and the market competition.

Variables	(1)	(2)	(3)	(4)
	GIS_t+1_	GIZ_t+1_	GIS_t+1_	GIZ_t+1_
ROBOT	0.1503*** (7.6886)	0.1450*** (8.3441)	0.1536*** (8.9265)	0.1554*** (10.1177)
SOE	− 0.0473 (− 0.6496)	− 0.0738 (− 1.1467)		
ROBOT*SOE	0.0434** (2.3495)	0.0608*** (3.5111)		
HHI			− 0.0640* (− 1.6700)	− 0.0565* (− 1.7202)
ROBOT*HHI			0.0295*** (2.8197)	0.0312*** (3.3337)
Constant	− 5.1346*** (− 7.6394)	− 4.1930*** (− 7.0406)	− 4.8101*** (− 7.2360)	− 3.7352*** (− 6.2783)
Controls	Yes	Yes	Yes	Yes
Industry FE	Yes	Yes	Yes	Yes
Year FE	Yes	Yes	Yes	Yes
Observations	14,831	14,831	14,831	14,831
R-squared	0.2390	0.2131	0.2388	0.2117

### The heterogeneity of the market competition

Due to increasing costs of raw materials, components, and other materials, along with weak bargaining power of enterprises in intense market competition, they lack sufficient pricing power^70^. As market competition becomes fiercer, enterprises are increasingly inclined to adopt industrial robots. Industrial robot adoption has the potential to improve labor productivity and increase investment in green technology and green products, thereby promoting green innovation, especially in enterprises operating in highly competitive markets. Therefore, we expect that industrial robot adoption can experience a more significant promotion in driving green innovation in enterprises facing intense market competition.

We employ the Herfindahl–Hirschman Index (HHI) to calculate the market competition degree. If HHI is less than the sample median, the value is 1; otherwise, it is 0. A smaller HHI value indicates a higher market competition degree. The interaction term between HHI and industrial robot adoption is constructed for regression. Columns (3) and (4) in Table 9 display the outcomes of the heterogeneity of market competition degree. The interaction term coefficients (ROBOT*HHI) remain significant at the 1% level. The outcomes demonstrate that in enterprises with intense market competition, industrial robot adoption yields a heightened influence on promoting corporate green innovation.

### The heterogeneity of carbon emissions intensity

Higher carbon emission regions are abundant in natural resources, resulting in high dependence of enterprises on natural resources and energy for their production and operation activities^71^. Moreover, higher carbon emission enterprises have a single energy structure, higher energy consumption and lower energy efficiency. Industrial robot adoption can enhance advanced energy conservation and emission reduction technologies, optimize energy efficiency, and decrease carbon emissions intensity^72,73^, thereby facilitating corporate green innovation. Consequently, we anticipate that industrial robot adoption can produce a prominent influence on green innovation in enterprises located in regions with higher carbon emissions.

Referring to Cheng et al.^74^, we employ two different definitions to measure carbon emissions intensity: the ratio of total carbon emissions to the total population (PEREMI) and the ratio of total carbon emissions to GDP (GEMI). If the carbon emissions intensity is above the median, the value is 1; otherwise, it is 0. The interaction term between carbon emissions intensity and industrial robot adoption is constructed for regression. Table 10 displays the regression outcomes, examining the heterogeneity of carbon emissions intensity. Notably, the coefficients associated with the interaction term (ROBOT*PEREMI) and the interaction term (ROBOT*GEMI) reach a significance level of 5%. These outcomes highlight that the effect of industrial robot adoption on the advancement of green innovation is more noticeable for enterprises located in regions characterized by higher carbon emissions.

**Table 10:** Heterogeneity analysis results of carbon emissions intensity.

Variables	(1)	(2)	(3)	(4)
	GIS_t+1_	GIZ_t+1_	GIS_t+1_	GIZ_t+1_
ROBOT	0.1348*** (8.2306)	0.1318*** (8.6226)	0.1505*** (7.6672)	0.1520*** (8.7312)
PEREMI	− 0.0040 (− 0.1758)	− 0.0206 (− 1.0224)		
ROBOT*PEREMI	0.0207** (2.1399)	0.0194** (2.0576)		
GEMI			0.0262 (0.7004)	− 0.0163 (− 0.5030)
ROBOT*GEMI			0.0349** (2.1085)	0.0363** (2.3743)
Constant	− 4.6466*** (− 7.1016)	− 3.7298*** (− 6.4071)	− 4.8952*** (− 7.3988)	− 3.7937*** (− 6.3830)
Controls	Yes	Yes	Yes	Yes
Industry FE	Yes	Yes	Yes	Yes
Year FE	Yes	Yes	Yes	Yes
Observations	14,831	14,831	14,831	14,831
R-squared	0.2339	0.2050	0.2395	0.2120

## Discussion

Firstly, industrial robots, as quintessential representatives of digital technology, are progressively emerging as a novel mode of production in the manufacturing industry, offering a pivotal avenue for advancing green transformation and upgrading of enterprises. This study uses data from Chinese listed companies between 2007 and 2019 to investigate the influence of industrial robot adoption on corporate green innovation and its underlying mechanisms. The findings underscore that industrial robot adoption significantly fosters corporate green innovation. Furthermore, this study dissects green innovation into both scale and quality facets, revealing that industrial robot adoption enhances both the scale and quality of corporate green innovation. Prior research predominantly delves into the impact of digital technology on corporate green innovation, often lacking in-depth exploration^75^. This study introduces industrial robots into the research framework, rendering the application of digital technology more concrete. This substantiates the affirmative role that industrial robots play in facilitating sustainable development of enterprise. Thus, from the perspective of listed companies, Chinese manufacturing enterprises can effectively harness industrial robots, offering valuable insights into elevating green technological innovation and environmental performance.

Secondly, in term of mediating effects, industrial robot adoption, by elevating corporate productivity and bolstering environmental management capabilities, fuels green innovation, thus indicating their role in “technology spillover” and “environmental governance effects”. In essence, other factors being constant, improvements in corporate productivity and environmental management capabilities stimulate green innovation. Concurrently, industrial robot adoption indirectly influences corporate green innovation through the mediation of productivity and environmental management capabilities. Our research results, unlike the perspective of Yuan and Pan^76^ regarding the impact pathways of digital technology on corporate green innovation, present a more comprehensive and integrated analysis. This study constructs a theoretical model and employs mediation analysis to scrutinize the impact of industrial robot adoption on corporate green innovation. From the perspectives of productivity and environmental management, it elucidates the underlying mechanisms of industrial robot adoption on corporate green innovation, thus providing empirical evidence to illuminate the “black box” of their interaction.

Lastly, with regard to moderating effects, this study examines the moderating role of industrial robots on corporate green innovation at both the enterprise and regional levels. The research reveals that in state-owned enterprises, enterprises with the intense market competition and enterprises in regions with higher carbon emissions intensity, the positive impact of industrial robot adoption on corporate green innovation is notably pronounced. Xu et al.^77^ indicate that state-owned enterprises positively mediate the relationship between digital transformation and corporate green innovation. This study further explores market competition and regional carbon emissions intensity factors. The conclusions not only deepen the scope of relevant research at both the corporate and regional levels but also enrich the analysis of interrelationships among these factors. In formulating pertinent policies, governments should consider the diversity at the enterprise and regional levels. Policies should be tailored to specific enterprise and industry characteristics to achieve breakthroughs in green innovation and energy conservation, thereby promoting enterprise green transformation and upgrading.

## Conclusion and policy implications

### Conclusion

As a key driver of innovation, artificial intelligence technology is a vital pathway to realize a green innovation and sustainable development model for enterprises. In this paper, we investigate how industrial robot adoption influences corporate green innovation and uncover the mechanisms of its effects regarding productivity and environmental management. By leveraging data from Chinese listed enterprises between 2007 and 2019, our findings demonstrate the significant positive effect of industrial robot adoption on corporate green innovation, bolstering both its quantity and quality. Moreover, the mechanism study shows that industrial robot adoption can enhance corporate productivity and environmental management capabilities, thereby fostering corporate green innovation. Furthermore, we further reveal the heterogeneous effects of industrial robots on green innovation. Specifically, industrial robot adoption can cause a more pronounced effect on facilitating green innovation in state-owned enterprises, enterprises with the intense market competition and enterprises in regions with higher carbon emissions intensity.

### Policy implications

Firstly, the government should implement relevant policies for intelligent manufacturing, vigorously promote the widespread adoption of artificial intelligence technology, and provide vital assistance to enterprises in their pursuit of sustainable development through technological methods. They should encourage enterprises to adopt industrial robots in environmental management, promote greater backing for green innovation, and guide the advancement of green technology through market mechanisms. Simultaneously, it is crucial for the government to grant enterprises substantial green subsidies and innovation funds. This will alleviate financial pressure, incentivize investments in environmental protection, and play the promoting role of artificial intelligence technology in driving green innovation within the manufacturing enterprises.

Secondly, enterprises should fully recognize the significance of green innovation and its profound implications for the attainment of sustainable development. By introducing industrial robots into operational processes, enterprises can continuously improve green production processes and green product quality, and optimize the strategic combination of green innovation. Additionally, enterprises should actively foster a seamless amalgamation of artificial intelligence technology with diverse factors associated with green innovation. This proactive integration enables the realization of intelligent green production systems and effective environmental governance. Consequently, such endeavors generate spillover effects that propel and foster the growth of green innovation.

Thirdly, the government should take into account the heterogeneity at the regional and enterprise levels and formulate relevant policies that align with the unique characteristics of enterprises and industries. For enterprises in regions with higher carbon emissions intensity, the government should support them in fully leveraging the technological spillover effects of industrial robots and adopting artificial intelligence technology to achieve breakthroughs in green innovation and emission reduction, ultimately driving the green transformation.

## Data Availability

Data will be made available on request. If someone requests the raw data from this study, you can contact Lin Liang.
